# Theoretical Analysis of the Cycle of Intimate Partner Violence: A Systematic Review

**DOI:** 10.1177/15248380241301781

**Published:** 2024-12-11

**Authors:** Brittney McCloud, Alhassan Abdullah

**Affiliations:** 1 Flinders University, Adelaide, SA, Australia; 2 Charles Sturt University, Albury-Wodonga, NSW, Australia

**Keywords:** intimate partner violence, IPV, cycle of violence, IPV transmission, social learning theory

## Abstract

Research on the intergenerational transmission of intimate partner violence (IPV), or the “cycle of violence,” from childhood exposure (G1) to the perpetration of IPV in adulthood relationships (G2), has increased over the past decades. The link between childhood exposure to interparental violence and future IPV perpetration is largely explained by postulations from social and psychological theories, such as social learning theory. These theories provide claims regarding IPV transmission pathways and the mechanisms underpinning the transmission. However, evidence from extant theory-informed studies on the cycle of violence has generated several null and counter-predictive findings, which raises questions about the nature of the theory-informed research, as well as methodological questions. This systematic review sought to analyze how existing studies applied specific theories to research IPV transmission, and the mechanisms underpinning these transmissions. Following the PRISMA procedure for systematic reviews, we reviewed empirical articles from five databases (*Scopus*, *Web of Science*, *CINAHL*, *Informit*, and *PubMed*) published between 1990 and 2024. Results from the 30 included studies were synthesized under three theoretical categories, namely: social, psychological, and cultural theories. Under each theoretical category, we found inconsistent evidence, lack of empirical examination of theory-driven variables, and reductionist approaches, in terms of how claims from the theories are applied in research on IPV transmission. Research guided by a singular theoretical framework presented inconsistencies compared to those applying a multi-theory approach. As a result, we recommend an integrated theoretical model that considers the comprehensive and transactional process/factors that underpin IPV transmission.

## Introduction

Intimate partner violence (IPV) is a serious public health issue predominantly affecting women (Meyer et al., 2023; Morrison et al., 2024; Warren et al., 2024). IPV encompasses a pattern of abusive behaviors among previous or current partners in a romantic or intimate relationship (Machado et al., 2024). About 1 in 3 women have been subjected to violence by a current or previous intimate partner and approximately 38% of women murdered are committed by intimate partners (World Health Organization, 2024). Researchers have established IPV as the prevalent form of violence against women (Meyer et al., 2023; Morrison et al., 2024; Warren et al., 2024). Estimates by Peterson et al. (2018) suggest that it will cost a country (such as U.S.) about $3.6 trillion to effectively address the lifetime economic cost of IPV. IPV is also associated with several adverse outcomes, including suicide, depression, anxiety, alcohol and substance abuse, chronic pain, injury, and most likely death (Coker et al., 2002; Stubbs & Szoeke, 2022). The economic cost and adverse outcomes associated with IPV make efforts to curb the occurrence of IPV *sine qua non*.

Among the key mechanisms to prevent IPV occurrence is to break the cycle that transmits and re-produces IPV intergenerationally. IPV is a private form of violence that predominantly occurs within the family setting (Beyer et al., 2015), and often in front of children (Henderson et al., 2023). It is well argued that children who witness IPV incidences are more likely to perpetrate IPV, and empirical examination and evidence on the transmission of IPV perpetration has been influenced by several social, psychological, and behavioral theories. Yet, despite systematic reviews acknowledging evidence of counter-predictive and null findings in several empirical findings on IPV transmission (Evans et al., 2022; Kimber et al., 2018), there is no systematic review that offers comprehensive theoretical insight into the empirical evidence on IPV transmission and the theories underpinning these studies.

A wide range of research looks at the effects of children witnessing violence between their parents, particularly focusing on the intergenerational transmission of IPV, and the potential pathways to explain the occurrence of transmission into their own adult intimate relationships (Muniz & Zavala, 2023; Smith-Marek et al., 2015; Weir et al., 2021). Many of these studies follow postulations from social learning theory (SLT; Bandura, 1977). However, even with studies underpinned by SLT, a sizable number of them have reported counter-predictive (outcomes contradictive to those predicted by a theory) and theoretical null findings (results showing no statistically significant relationship between variables; Li et al., 2019; Smith-Marek et al., 2015). Both the counter-predictive and theoretical null findings raise questions about the viability of the theoretical logic and call for further investigation to unpack the theoretical statement logic. A failure to critically unpack the theoretical underpinnings of these studies could be detrimental to our understanding of IPV transmission, and how we study the pathways through which it transmits. This systematic review seeks to provide comprehensive evidence on the transmission pathway of IPV from one generation (G1, witnessing IPV as a child) to another generation (G2, perpetrating IPV as an adult) by analyzing the theories that underpin the studies and associated empirical evidence generated. This review is guided by two research questions:

(1) What theories underpin research on IPV transmission from witnessing IPV as a child (G1) and perpetrating IPV as an adult (G2).
(2) What is the relationship between the theoretical postulations and empirical evidence on the intergenerational transmission of IPV.

### Theory and Analysis

A dominant theoretical perspective used within research to explain the intergenerational transmission of IPV is social learning theory (SLT). Bandura’s (1977) SLT is predominantly acknowledged and cited throughout literature in providing an explanation for this phenomenon. SLT suggests that the majority of a child’s learning experiences relating to social interactions, and the formation of values, beliefs, attitudes, and behaviors are learned through observing and imitating interactions within their family environment (Bandura, 1977; Chernyak et al., 2020; Copp et al., 2019; Evans et al., 2022).

Akers enhanced SLT by developing the SLT of deviant behavior, using key elements of both Edwin Sutherland’s differential association theory and Bandura’s (1977) SLT. Akers (1977) SLT identifies the core constructs of this theory as differential association, differential reinforcement, imitation or modeling, and definitions. Akers provided a broad framework for understanding the social processes underlying deviant behavior emphasizing both social reinforcements of behaviors and imitation in the learning process. SLT provides valuable insight into how behaviors are learned through the process of observational learning and modeling (Copp et al., 2019; Valgardson & Schwartz, 2019), thus providing reasonable grounds in the justification of how IPV transmits intergenerationally.

However, it could be argued that SLT itself encompasses a reductionist approach by oversimplifying the multifaceted nature and complexities involved with IPV transmission, which presents limitations in understanding the process of IPV transmission. The intergenerational transmission of IPV cannot be narrowed down or reduced to simply being observational learning as there is strong evidence of key determinants, such as childhood experiences of trauma (Whitten et al., 2024), broader sociocultural contexts, and attachment styles within the family context (Li et al., 2019). Additionally, interpreting IPV transmission via SLT only overlooks the agency and cognitive capacity of children.

Consistent with SLT postulations, in a family with more than one child, it could be assumed that all children exposed to interparental violence will normalize and accept violent behaviors (Copp et al., 2019) as a method for managing conflict or gaining power and control within an intimate relationship (Li et al., 2019). These children will then be expected to perpetrate violence as adults. However, there is no specific evidence to determine whether all children in the family will indeed go on to be violent. It could be the case that while one child normalizes and accepts acts of violence to gain control in conflictual situations and goes on to become a perpetrator of IPV, their sibling/s may not consider such behaviors as an appropriate means for managing conflict. It is therefore safe to argue that the link between witnessing IPV and perpetrating IPV is complex and multifarious, and evidence on the transmission pathway has not been fully explored due to the heavy reliance on SLT which may not provide a full account of the transmission cycle. Shakoor et al. (2022) argue there are inconsistencies within the existing evidence and the theories explaining IPV transmission, which limits the capacity to examine the processes that underpin the perpetration of violence in adult intimate relationships.

A continued reliance on SLT for studying IPV transmission oversimplifies the complexities of behavioral aspects of IPV and attempts to narrow the IPV transmission processes down to simple imitation. If it were simply imitation, future behavior of children exposed to interparental IPV would present like-for-like scenarios in their own adult relationships. Rather, we posit that psychological, sociological, and cultural theories must be considered when studying IPV transmission. For example, Durkheim (1893) highlights the sociological processes that reinforce behaviors, including the presence or absence of social control, and punishment. This systematic review adopts a holistic and eclectic theoretical approach to unpack the transmission of IPV from G1 to G2 focusing on the psychological, cultural, and sociological theories and the evidence generated from studies underpinned by some of these theories.

### Pathways of IPV Transmission: Predictive Factors

During childhood, family is the main source of social interaction. Children’s values, beliefs, and attitudes are typically formed during the early years based on observations made within their social environment (Bandura, 1977). Children who grow up in a household where IPV occurs between parents are at increased risk of witnessing such behaviors (Valgardson & Schwartz, 2019). Exposure to interparental violence is acknowledged as a predictive pathway to the intergenerational transmission of IPV (Adams et al., 2019; Low et al., 2019; Shakoor et al., 2022). However, various complex factors have been identified that can influence the likelihood of IPV perpetration among children who have been exposed to IPV in their childhood.

There is evidence to suggest that a history of witnessing or experiencing child maltreatment is a predictive outcome for IPV perpetration in adulthood (Adams et al., 2019; Cascardi, 2016; Low et al., 2019; Powers et al., 2020). Additionally, some research claims that exposure to interparental IPV is a form of child maltreatment itself (Adams et al., 2019). During childhood, repeated exposure to violent behaviors through direct experience or witnessing, increases the chance of normalizing and accepting violent or aggressive behaviors (Shakoor et al., 2022) in responding to and managing conflictual situations (Powers et al., 2020; Valgardson & Schwartz, 2019; Warren et al., 2024). While the hypothesis of “violence begets violence” is supported within some empirical studies (Cascardi, 2016; Copp et al., 2019; Muniz & Zavala, 2023), others have identified inconsistencies in such findings (Li et al., 2019; Smith-Marek et al., 2015). Evidence remains weak in identifying a direct correlation between experiencing child maltreatment and exposure to interparental IPV as a predictive outcome that guarantees IPV perpetration in adult intimate relationships.

Another well-recognized transmission pathway of IPV is gendered modeling. The gender-specific model hypothesizes that a child is more inclined to imitate behaviors witnessed by the same-sex parent (Evans et al., 2022; Li et al., 2019; Shakoor et al., 2022). In theory, a son witnessing violence perpetrated by their father toward their mother provides a predictive basis for that child to perpetrate violence toward an intimate partner in later years (Hou et al., 2016; Morrison et al., 2024). The gender-specific modeling theory was supported by Lee et al. (2013) study in the United States, in which they concluded that males witnessing interparental IPV were at increased risk for normalizing violent interactions within intimate relationships. Evidence from Machado et al. (2024) suggests that children who witness bidirectional interparental violence (IPV perpetrated by both mother and father) are much more likely to perpetrate IPV later in life.

### Gaps in the Literature and Justification for a Systematic Review

Though several factors and mechanisms may explain the intergenerational transmission of IPV, critical theoretical gaps remain within the literature. While psychological processes associated with IPV transmission have been explored within existing literature, findings are inconsistent with theories used thus far. Likewise, SLT is frequently cited to explain IPV transmission via the theoretical logic of behavioral imitation; however, this explanation could be reductionist as it oversimplifies the multifaceted nature of IPV, and the transmission of IPV intergenerationally. Consequently, the purpose of conducting this systematic review is to coalesce existing empirical evidence of the transmission of IPV intergenerationally by analyzing the theoretical postulations and the empirical evidence generated through these studies. The review will offer significant evidence and insight to guide future research on IPV transmission and theory development.

## Methods

### Search Strategy

Literature searches were conducted from January to March 2024 in five databases: *CINAHL*, *Informit*, *Scopus*, *Web of Science*, and *PubMed*. An initial pilot search to test keywords relating to the intergenerational transmission of IPV was conducted prior to the overall search of electronic databases. The online database search was not restricted to any limitations (such as year, article type, etc.) to ensure all results could be extracted for thorough screening. The search was conducted using a combination of topic keywords developed in consultation with a University librarian, and Boolean operators (such as AND/OR) were included. Exact search terms used across all databases included: IPV OR “intimate partner violence” OR “domestic violence” OR “family violence” OR “interparental violence” AND “child exposure” OR “child witness*” OR “child experience” OR victimization* AND intergenerational OR generational OR transmission OR perpetration.

The entire process was guided by the Preferred Reporting Items for Systematic Review (PRISMA). Prior to conducting a database search, the researchers reviewed the PROSPERO platform to ensure that no duplicate studies were currently being conducted. Subsequently, the review was registered in PROSPERO (registration ID: CRD42024537412).

### Inclusion and Exclusion Criteria

To be eligible for inclusion in this systematic review, studies were expected to: (a) include evidence generated mainly through research with children, adolescents, and adults of any gender with exposure to IPV in childhood (0–17 years) as a key variable; (b) report perpetration of IPV behaviors within an intimate relationship in the young adulthood period or adulthood; (c) have hypotheses informed explicitly by a theory or a combination of theoretical postulations; and (d) published using the English language to ensure readability. No geographical limitations were applied to ensure the scope of literature captured what is known about the subject globally. Any method of study design was included for screening.

Articles were excluded if they were (a) published before 1990; (b) not peer-reviewed (e.g., theses, dissertations, books, book chapters, commentaries, editorials, etc.); (c) focused on child sexual abuse or other forms of child maltreatment, delinquent or deviant behavior, substance abuse, etc.; (d) did not include evidence of exposure to interparental violence; and (e) did not mention a specific theory that informed the study.

### Study Selection and Screening

**Figure 1.**
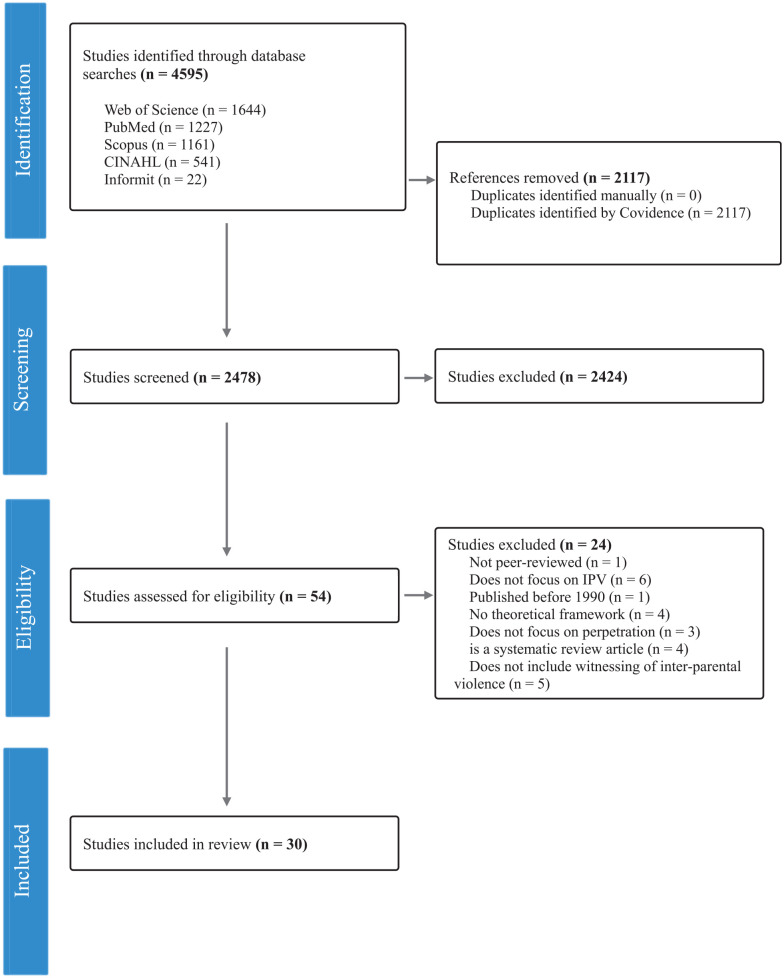
PRISMA flow diagram. PRISMA = preferred reporting items for systematic review.

The initial searches resulted in 4,595 articles. Results were downloaded to Covidence, and 2,117 duplicate articles were removed. The remaining 2,478 articles were independently screened by one reviewer by title and abstract against the eligibility criteria. Titles and abstracts that included key terms (e.g., intergenerational transmission, cycle of violence, witnessing intimate partner violence, family-of-origin) and met inclusion criteria were included for full-text review. Fifty-four articles were deemed eligible for full-text screening and were reviewed independently by two researchers, with a further 17 articles excluded for not satisfying the scope of the study. The remaining 37 articles were read thoroughly, and an additional seven articles were removed for not including a theoretical framework or not having a focus on witnessing parental IPV. The screening process is presented in the PRISMA diagram below (Figure 1).

### Data Extraction

A data extraction template was created on covidence to extract key information for each included article. Information extracted included publication details (e.g., author, year of publication, country), study design (e.g., cross-sectional and longitudinal), sample characteristics (e.g., sample size), study details (e.g., objectives/aims and form of IPV exposure measured), theory (e.g., SLT, and attachment theory), and finally outcomes and key findings. Articles deemed eligible for data extraction and synthesis were independently reviewed by one reviewer (first author) and verified by the second reviewer to ensure the validity of extraction results. Detailed results and findings from the included studies were extracted for further analysis and synthesis.

### Data Synthesis and Analysis

A separate Word document was created for each of the included studies, with each Word document containing evidence from one study. Information included in the Word document was author names, study objective, hypothesis, theories informing the study, summary methodology, and key findings. The key findings section included narrative evidence of the associations examined, regression coefficients, and associated *p*-values. We then organized the findings under core theories that informed the study. Theories, such as trauma, and SLT, and the associated evidence were classified under two broad theoretical categories, social and psychological theories. Under each classification, results were sub-grouped based on the specific theories mentioned in the included studies. Nuances pertaining to culture and gender were also evident in the included studies and thus organized as the third and fourth classifications. A detailed report of the results has been presented under the four broad classifications in the results section.

### Quality Appraisal

We used the Mixed Methods Appraisal Tool Version 2018 (MMAT) (Hong et al., 2018) to assess the quality of the included studies. The MMAT includes methodological quality criteria checks for qualitative, quantitative, and mixed methods studies by asking two main screening questions for all types: (S1) Are there clear research questions, and (S2) Do the collected data allow to address the research questions (Hong et al., 2018). A further five appraisal questions are categorized by methodological study design and scored based on the fulfillment of the MMAT criteria. The appraisal showed that all included studies were of high quality as all key elements were reported in most of the studies (see Table 1). We did not calculate overall appraisal scores as the MMAT discourages calculating an overall numerical score based on the ratings of each criterion (Hong et al., 2018). Furthermore, excluding studies with a low methodological quality score is discouraged as the MMAT does not determine the appropriateness of inclusion (Hong et al., 2018).

**Table 1.** Characteristics of Included Studies.

Author/s, (Year of Publication)	Study Design, (Country)	Sample Size	Objective/Aims	Theory	Form of IPV exposure (Scales)	Outcome/Key Findings
Antle et al. (2020)	Cross-sectional (United States)	*n* = 9	To explore the impact of family-of-origin violence on adolescent attitudes in their romantic relationships	Social learning theory, attachment theory	Physical violence; verbal abuse; coercive control	Adolescents exposed to IPV in their family of origin were more accepting of violence in their dating relationships
Cheung and Huang (2023)	Longitudinal (United States)	*n* = 685	To examine childhood exposure to IPV and the involvement in teen dating violence among adolescents	Trauma theory; social learning theory	Physical violence; emotional control; economic abuse; child neglect (CTS2, CTSPC)	Children exposed to IPV are more likely to have greater teen dating violence involvement
Craft and Serovich (2005)	Cross-sectional (United States)	*n* = 25	To explore the incidence of partner abuse of gay men who were HIV positive	Social learning theory; ecological theory	Psychological aggression; physical assault; sexual abuse; injury; parent–child violence (CTS2, FOVS)	Partial support for family of origin violence contributing to subsequent violence in intimate relationships
Eriksson and Mazerolle (2015)	Cross-sectional (United States)	*n* = 303	To examine the association between exposure to violence in childhood and perpetration of IPV in adulthood among males	Social learning theory	Physical violence through father-to-mother; mother-to-father; bidirectional (Family Violence Scale)	Men exposed to interparental violence and experiencing child abuse were more likely to perpetrate IPV in adulthood
Fergusson et al. (2006)	Longitudinal (New Zealand)	*n* = 1,265	To examine exposure to interparental violence in predicting later involvement in IPV	Psychosocial	Physical violence; verbal abuse; psychological abuse; threaten with a weapon (CTS, CTS2)	Weak linkage between exposure to domestic violence in childhood and violent behaviors in adulthood
Forke et al. (2018)	Cross-sectional (United States)	*n* = 907	To explore the relationship between witnessing violence perpetrated by adults and subsequent IPV victimization and/or perpetration in adolescent relationships	Social learning theory; attachment theory	Physical violence; emotional abuse; sexual violence (CTS2)	Childhood witnessing of adult violence at home is an indicator of adolescent relationship violence
Forke et al. (2021)	Cross-sectional (United States)	*n* = 907	To measure associations between exposure to childhood witnessing of adult violence at home and later involvement with adolescent relationship violence	Social learning theory; attachment theory	Psychological aggression; physical assault; sexual abuse; injury (CTS2, CADRI)	Witnessing violence during childhood increased experience of victimization and perpetration in adolescent relationships
Franklin et al. (2012)	Cross-sectional (United States)	*n* = 439	To investigate the relationship between family-of-origin violence histories and the risk and resiliency factors that mediate the effect of IPV	Intergenerational transmission of violence theory; social learning theory	Physical violence; verbal abuse; psychological abuse (CTS2)	Cumulative effects of multiple forms of violence (i.e., corporal punishment and witnessing interparental violence) significantly affected adult IPV perpetration
Fritz et al. (2012)	Cross-sectional (United States)	*n* = 453 couples	To examine four forms of family-of-origin aggression and subsequent experience of physical IPV in adulthood	Social learning theory; intergenerational transmission of violence theory	Psychological aggression; physical assault; sexual abuse; injury; parental violence; parent–child violence (CTS2, FOA)	Children who are exposed to multiple forms of violence (mother-to-child, interparental violence exposure) have a larger influence on IPV perpetration during adulthood
Gover et al. (2008)	Cross-sectional (United States)	*n* = 2,298–2,305 valid samples	To examine the relationship between experiencing and perpetrating dating violence and exposure to violence in the family of origin	Intergenerational transmission of violence theory	Physical violence; verbal abuse; psychological abuse (CTS2)	Witnessing parental violence does not have a significant impact on dating violence perpetration, however, females who witnessed paternally perpetrated abuse had significant rates of dating victimization
Haselschwerdt et al. (2021)	Cross-sectional (United States)	*n* = 23 women	To examine the nature of domestic violence exposure during childhood and young adults’ intimate relationships	Social learning theory; intergenerational transmission of violence theory	Physical violence; verbal abuse; financial abuse; coercive control	Exposure to violence in the family of origin increased the risk of victimization in intimate relationships
Hernández and Durán (2021)	Cross-sectional (Peru)	*n* = 74,204 women	To examine the effect personal history of violence has on the likelihood of IPV against women	Intergenerational transmission of violence theory	Physical violence; psychological violence; sexual violence (CTS)	Witnessing interparental violence produces a persistence effect on the intergenerational transmission of violence
Heyman and Smith Slep (2002)	Cross-sectional (United States)	*n* = 6,002	To analyze the exposure of various forms of family-of-origin violence and the risk for adult family violence perpetration	Intergenerational transmission of violence theory	Physical violence; verbal abuse; psychological abuse; threaten with a weapon (CTS)	Exposure to father-to-mother violence increased men’s family violence perpetration and women’s victimization
Jung et al. (2019)	Longitudinal (United States)	*n* = 326	To examine gender differences in the prediction of IPV with a history of child abuse and exposure to parental IPV	Social learning theory	Physical violence; psychological aggression; sexual coercion; injury (CTS, CTS2)	Experiencing physical-emotional child abuse and domestic violence exposure predicted a higher likelihood of multitype violence for males
Karlsson et al. (2016)	Cross-sectional (United States)	*n* = 918	To examine the association between witnessing interparental violence and greater acceptance of violence in predicting teen dating violence victimization	Social learning theory	Physical violence; psychological aggression; injury (CTS, ACV, CADRI)	Witnessing violence in the family of origin, acceptance of dating violence, and acceptance of female perpetrated violence were significant predictors of both psychological and physical teen dating violence victimization
Kerley et al. (2010)	Cross-sectional (Thailand)	*n* = 793	To examine how exposure to interparental violence in childhood is related to IPV perpetration and victimization during adulthood	Social learning theory; intergenerational transmission of violence theory	Physical violence; verbal abuse; psychological abuse (CTS, CTS2)	Witnessing interparental violence is both directly and indirectly associated with future physical victimization (but not perpetration) for Thai women
Kernsmith (2006)	Cross-sectional (United States)	*n* = 114	To examine the impact of family-of-origin violence on future violence perpetration in intimate relationships	Social learning theory; intergenerational transmission of violence theory	Physical violence; verbal abuse; psychological abuse; sexual abuse (CTS, PMWI)	Exposure to and experiencing abuse in the family of origin has profound impact on experiences in adult relationships
Knight et al. (2016)	Longitudinal (United States)	*n* = 313	To examine the continuity of IPV across respondents’ life course and into the next generation	Social learning theory; interactional life course theories	Physical violence; verbal abuse; psychological abuse; threaten with a weapon (CTS)	There is evidence for the continuity of IPV over the life course rather than intergenerational continuity
Kwon and You (2023)	Cross-sectional (South Korea)	*n* = 526	To examine the gender and role associations between domestic violence and dating violence victimization in a sample of male college students	Social learning theory; Intergenerational transmission of violence theory	Physical violence; verbal abuse; psychological abuse; threaten with a weapon (CTS, CTS2, JVS)	Results support the gender and role-specific hypotheses in that children model the behavior (perpetration or victimization) of the same-sex parent
Kwong et al. (2003)	Cross-sectional (Canada)	*n* = 1,249	To test three social learning models of cross-generational violence (general modeling hypothesis, role-specific, and gender specific)	Social learning theory	Physical violence; verbal abuse; psychological abuse; threaten with a weapon (CTS2, PMWI)	Exposure to family-of-origin violence was associated with a greater likelihood of perpetration and victimization in
Mehfooz et al. (2023)	Cohort (31 countries)	*n* = 466,330 women	To assess the effect of women’s childhood exposure to interparental violence and their attitudes toward IPV in their adult life	Social learning theory; intergenerational transmission of violence theory	Physical abuse	Women who witnessed father-to-mother violence were more likely to justify partner violence; violence becomes transmitted intergenerationally (supporting SLT)
Menard et al. (2014)	Longitudinal (United States)	*n* = 1,281	To examine the relationships between exposure to violence (interparental, community) to perpetration and victimization of IPV in adulthood	Social learning theory; general strain theory	Physical violence; verbal abuse; psychological abuse; threaten with a weapon (CTS)	For males, only physical abuse remains significant as a predictor. For females, adolescent exposure to violence is not predictive of adult IPV perpetration or victimization
Morris et al. (2015)	Longitudinal (United Kingdom)	*n* = 461	To examine the links between exposure to family violence in pre-adolescence and pro-violent beliefs or behaviors in early adolescence and dating violence in late adolescence	Social learning theory	Psychological aggression; physical assault; sexual abuse; injury (CTS, CADRI, CPIC)	Aggressive behaviors and peer deviance in early adolescence may contribute directly to late adolescent dating violence perpetration and victimization
Nam and Maxwell (2020)	Cross-sectional (South Korea)	*n* = 330	To examine the direct effects of witnessed parental conflict and guilt on dating violence	Social learning theory; intergenerational transmission of violence theory	Physical violence; emotional abuse; parent–child violence (FOA, witnessing domestic violence measure)	Guilt moderates the effect of witnessed parental conflict on emotional but not physical violence
Nikulina et al. (2021)	Cross-sectional (United States)	*n* = 284	To examine the individual and cumulative associations between nine adverse childhood experiences and IPV in emerging adulthood	Social learning theory; intergenerational transmission of violence theory	Physical violence; verbal abuse; psychological abuse; threaten with a weapon (CTS2, ACE’s)	Witnessing domestic violence was significantly associated with perpetration and victimization of physical aggression and injury
Ruel et al. (2020)	Longitudinal (Canada)	*n* = 2,564	To investigate gender differences in the relationships between exposure to interparental violence and acceptance of dating violence	Social learning theory; intergenerational transmission of violence theory	Physical violence; verbal abuse; psychological abuse; threaten with a weapon (CTS2, CADRI, self-efficacy to deal with violence scale, acceptance of prescribed norms scale)	Exposure to interparental violence was linked to acceptance of girl-perpetrated violence and victimization among both genders
Shields et al. (2020)	Cross-sectional (Canada)	*n* = 22,066	To examine associations between gender, three types of child maltreatment and victimization of IPV in adulthood	Ecological systems theory; social learning theory	Physical violence; verbal abuse; psychological abuse; threaten with a weapon (CTS2, CEVQ)	Child maltreatment and exposure to IPV were associated with IPV in adulthood for both sexes
Sokar et al. (2023)	Cross-sectional (Israel)	*n* = 604	To examine the mediating role of attachment insecurities and gender differences in the relationship between childhood exposure to parental violence and IPV perpetration during adulthood	Social learning theory; attachment theory	Psychological aggression; physical assault; sexual abuse; injury; parental violence; parent–child violence (CTS2, CTSPC, ECR-R)	Exposure to physical violence is a risk factor for IPV perpetration
Wareham et al. (2009)	Cross-sectional (United States)	*n* = 204	To examine the direct and indirect transmission of family-of-origin violence by applying social learning theory and the intergenerational transmission of violence theory	Social learning theory; intergenerational transmission of violence theory	Physical violence; verbal abuse; psychological abuse; threaten with a weapon (CTS, FOVS)	A combined model of social learning theory and intergenerational transmission theory provides a comprehensive explanation of minor and severe IPV prevalence among men
You and Kwon (2023)	Cross-sectional (South Korea)	*n* = 550	To investigate the relationship between domestic violence and dating violence victimization and the mediating influence of the justification of dating violence	Social learning theory; intergenerational transmission of violence theory	Physical violence; verbal abuse; psychological abuse; threaten with a weapon (CTS, CTS2, JVS)	Witnessing interparental violence and experiencing child abuse influenced dating violence victimization

*Note*. ACE = adverse childhood experiences survey; ACV = acceptance of couple violence scale; CADRI = conflict in adolescent dating relationships inventory; CEVQ = childhood experiences of violence questionnaire; CPIC = children’s perception of interparental conflict scale; CTS = conflict tactics scale; CTS2 = revised conflict tactics scale; CTSPC = parent-child conflict tactics scales; ECR-R = experiences in close relationships revised; FOA = family of origin aggression scale; FOVS = the family of origin violence scale; IPV = intimate partner violence; JVS = justification of violence scale; PMWI = psychological maltreatment of women inventory; SLT = social learning theory.

## Results

### Descriptive Analysis of Study Characteristics

A total of 30 studies were included in this review. Twenty-two (73%) of the studies were cross-sectional with a total of 113,215 participants across all studies. Seven (23%) studies were longitudinal in nature with a collective sample size of 6,895 participants, including children, adolescents, and parents (or adults). One cohort study that collected data from 31 countries included a sample size of 466,330 women.

The studies included for synthesis were conducted across eight different countries and one cross-continental cohort study including 31 countries, presenting broad global data. Nineteen (63%) studies were conducted in the United States (*cf*. Antle et al., 2020; Cheung & Huang, 2023; Craft & Serovich, 2005; Eriksson & Mazerolle, 2015; Forke et al., 2018, 2021; Franklin et al., 2012; Fritz et al., 2012; Gover et al., 2008; Haselschwerdt et al., 2021; Heyman & Smith Slep, 2002; Jung et al., 2019; Karlsson et al., 2016; Kernsmith, 2006; Menard et al., 2014; Nikulina et al., 2021; Wareham et al., 2009). Three studies were conducted each in South Korea (Kwon & You, 2023; Nam & Maxwell, 2021; You & Kwon, 2023), and Canada (Kwong et al., 2003; Ruel et al., 2020; Shields et al., 2020), and one each from U.K. (Morris et al., 2015), New Zealand (Fergusson et al., 2006), Thailand (Kerley et al., 2010), Peru (Hernández & Durán, 2021), and Israel (Sokar et al., 2023). Mehfooz et al. (2023) cohort study included a cross-continental sample of 466,330 women from 31 countries in Africa and Asia. Broadly, all 30 articles focused on the intergenerational transmission of IPV with varying parameters for samples. A summary of the findings and characteristics of each of the included studies can be found in Tables 2–4.

**Table 2.** Quality Appraisal Table (MMAT).

Qualitative	S1	S2	1.1	1.2	1.3	1.4	1.5
Antle et al. (2020)	Y	Y	Y	Y	Y	Y	Y
Haselschwerdt et al. (2021)	Y	Y	Y	CT	Y	Y	Y
Quantitative	S1	S2	4.1	4.2	4.3	4.4	4.5
Cheung and Huang (2023)	Y	Y	Y	Y	Y	N	Y
Craft and Serovich (2005)	Y	Y	Y	Y	Y	Y	Y
Eriksson and Mazerolle (2015)	Y	Y	Y	Y	Y	Y	Y
Fergusson et al. (2006)	Y	Y	Y	Y	Y	Y	Y
Forke et al. (2018)	Y	Y	Y	Y	Y	Y	Y
Forke et al. (2021)	Y	Y	Y	Y	Y	Y	Y
Franklin et al. (2012)	Y	Y	Y	Y	Y	Y	Y
Fritz et al. (2012)	Y	Y	Y	Y	Y	CT	Y
Gover et al. (2008)	Y	Y	Y	Y	Y	Y	Y
Hernández and Durán (2021)	Y	Y	Y	Y	Y	Y	Y
Heyman and Smith Slep (2002)	Y	Y	Y	Y	Y	N	Y
Jung et al. (2019)	Y	Y	Y	Y	Y	Y	Y
Karlsson et al. (2016)	Y	Y	Y	Y	Y	Y	Y
Kerley et al. (2010)	Y	Y	CT	Y	Y	Y	Y
Kernsmith (2006)	Y	Y	Y	Y	Y	CT	CT
Knight et al. (2016)	Y	Y	Y	Y	Y	N	Y
Kwon and You (2023)	Y	Y	Y	Y	Y	Y	Y
Kwong et al. (2003)	Y	Y	Y	Y	Y	N	Y
Mehfooz et al. (2023)	Y	Y	Y	Y	N	Y	Y
Menard et al. (2014)	Y	Y	Y	Y	Y	N	Y
Morris et al. (2015)	Y	Y	Y	Y	Y	Y	Y
Nam and Maxwell (2020)	Y	Y	Y	Y	Y	Y	Y
Nikulina et al. (2021)	Y	Y	Y	Y	Y	N	Y
Ruel et al. (2020)	Y	Y	Y	Y	Y	N	Y
Shields et al. (2020)	Y	Y	Y	Y	Y	N	Y
Sokar et al. (2023)	Y	Y	Y	Y	Y	N	Y
Wareham et al. (2009)	Y	Y	Y	Y	Y	Y	Y
You and Kwon (2023)	Y	Y	Y	Y	Y	Y	Y

Note: Y = yes, N = no, CT = can’t tell, MMAT = Mixed Methods Appraisal Tool.

Items: Screening questions (for all types): S1) Are there clear research questions; S2) Do the collected data allow to address the research questions. Qualitative: 1.1) Is the qualitative approach appropriate to answer the research question; 1.2) Are the qualitative data collection methods adequate to address the research question; 1.3) Are the findings adequately derived from the data; 1.4) Is the interpretation of results sufficiently substantiated by data; 1.5) Is there coherence between qualitative data sources, collection, analysis, and interpretation. Quantitative descriptive: 4.1) Is the sampling strategy relevant to address the research question; 4.2) Is the sample representative of the target population; 4.3) Are the measurements appropriate; 4.4) Is the risk of nonresponse bias low; 4.5) Is the statistical analysis appropriate to answer the research question.

**Table 3.** Critical Findings.

• Majority of empirical research on the cycle of violence is informed by postulations from the social learning theory yet evidence is often counter predictive or null.
• Existing theoretical frameworks present a reductionist approach in explaining the transmission of IPV and fail to acknowledge the complex processes involved in transmission of violence behaviors.
• The interaction of social, psychological, behavioral, and cultural processes should be considered when examining the cycle of IPV transmission.

*Note*. IPV = intimate partner violence.

**Table 4.** Implications for Research, Theory, and Practice.

Research	• Future research should examine theory-driven variables in order to assess the mechanisms that motivate the transmission of IPV from witnessing IPV as a child to perpetrating IPV in adulthood. • Future research on IPV transmission should consider the use of multi-theory analysis. • Research that explores the transactional and interactional relationship between cultural, social, psychological, and identity factors will be desired to provide accurate evidence on the transmission of IPV.
Theory and practice	• Development of a comprehensive integrated theoretical framework could provide better insight into the processes involved in the intergenerational transmission of IPV. • Developing interventions targeted toward children exposed to interparental IPV should consider aspects of attachment theory. Providing adequate support in forming secure attachments may reduce the incidence of IPV transmission intergenerationally.

*Note*. IPV = intimate partner violence.

#### Samples

Majority of the studies included both male and female participants (*n* = 21). Four (13%) comprised of male only participants including gay men with HIV (Craft & Serovich, 2005), arrestees in a drug abuse monitoring program (Eriksson & Mazerolle, 2015), undergraduate university students (Kwon & You, 2023), and registered adult male batterers in a domestic violence program (Wareham et al., 2009). The remaining five (16%) comprised of female only participants, including mothers (Cheung & Huang, 2023), college students (Haselschwerdt et al., 2021), married women (Kerley et al., 2010), and two included women respondents of health surveys (Hernández & Durán, 2021; Mehfooz et al., 2023).

Notably, teen dating violence (TDV) was examined within five of the included studies. These studies focused on issues such as involvement in TDV after exposure to IPV in early childhood (Cheung & Huang, 2023), attitudes toward and acceptance of dating violence (Antle et al., 2020; Karlsson et al., 2016), pro-violent beliefs (Morris et al., 2015), and self-efficacy to disclose TDV (Ruel et al., 2020) in their own adolescent romantic relationships.

#### Measures of IPV Exposure

The studies included 28 quantitative and two qualitative articles. Various scales were applied within the included studies to measure child exposure to IPV, with many of the studies including one or more scales in their process of measurement. The conflict tactics scale (CTS) and revised conflict tactics scale (CTS2) were most applied within studies (*n* = 24) with varying modifications to measure forms of physical, psychological, and coercive forms of abuse in intimate relationships. Four studies (Forke et al., 2021; Karlsson et al., 2016; Morris et al., 2015; Ruel et al., 2020) utilized the Conflict in Adolescent Dating Relationships Inventory (CADRI) to measure the complexity of dating violence that occurs within adolescent intimate relationships.

Scales to measure justification and acceptance of violence included: Acceptance of Couple Violence Scale (Karlsson et al., 2016), Justification of Violence Scale (Kwon & You, 2023; You & Kwon, 2023), and Acceptance of Prescribed Norms Scale (Ruel et al., 2020). Various scales (*n* = 8) were applied to measure child and family experiences of violence: Family of Origin Aggression Scale (Fritz et al., 2012; Nam & Maxwell, 2021), The Family of Origin Violence Scale (Craft & Serovich, 2005; Wareham et al., 2009), Family Violence Scale (Eriksson & Mazerolle, 2015), Parent–Child Conflict Tactics Scale (Cheung & Huang, 2023; Sokar et al., 2023), Childhood Experiences of Violence Questionnaire (Shields et al., 2020), Adverse Childhood Experiences Survey (Nikulina et al., 2021), Children’s Perception of Interparental Conflict Scale (Morris et al., 2015), and Witnessing Domestic Violence measure (Nam & Maxwell, 2021).

Four additional scales and questionnaires were utilized to measure forms of violence and aggression, these included: Forms of Aggression Questionnaire (Nam & Maxwell, 2021), Self-Efficacy to Deal with Violence Scale (Ruel et al., 2020), Psychological Maltreatment of Women Inventory (PMWI) (Kernsmith, 2006; Kwong et al., 2003), and Experiences in Close Relationships-Revised (Sokar et al., 2023). Finally, three studies (Antle et al., 2020; Haselschwerdt et al., 2021; Mehfooz et al., 2023) did not explicitly state the use of scales within their study but they formulated operational questions relating to forms of violence witnessed based on their hypothesis.

#### Theoretical Frameworks

All studies included for synthesis in this systematic review explicitly stated one or more theoretical perspectives to formulate hypotheses. Twenty-six of the 30 included studies applied SLT, and fifteen studies were informed by the intergenerational transmission of violence theory. Other theoretical perspectives included were attachment theory (Antle et al., 2020; Forke et al., 2018, 2021; Sokar et al., 2023), trauma theory (Cheung & Huang, 2023), ecological theory (Craft & Serovich, 2005; Shields et al., 2020), psychosocial theory (Fergusson et al., 2006), general strain theory (Menard et al., 2014), and interactional life course theories (Knight et al., 2016). The remaining parts of this section will present evidence of the theoretical frameworks that guided the studies and how they were applied to explain the cycle of violence.

### Thematic Synthesis of Theoretical Predictions and Empirical Results

#### Social Theories

Social theories are used to explore and interpret social phenomena. These theories provide explanations for social behaviors, relationships, and how various aspects of society and social life may influence social change. For this review, the theoretical frameworks categorized into social theories are: SLT, ecological theory, intergenerational transmission of violence, psychosocial theory, and interactional theory.

Bandura’s (1977) SLT postulates that children who witness parental violence in their family-of-origin go on to model or mimic such violent behaviors (Sokar et al., 2023) and have greater acceptance (Antle et al., 2020; Karlsson et al., 2016) for violence in their own intimate relationships. Twenty-six of the included articles applied SLT to inform their research. More than half of the studies (*n* = 14) testing hypothesis informed by SLT found consistent significant results on the relationship between exposure to IPV and perpetration of IPV in adult relationships (see, Cheung & Huang, 2023; Forke et al., 2021; Karlsson et al., 2016; Kerley et al., 2010; Kernsmith, 2006; Knight et al., 2016; Kwon & You, 2023; Kwong et al., 2003; Mehfooz et al., 2023; Morris et al., 2015; Nam & Maxwell, 2021; Ruel et al., 2020; Sokar et al., 2023; You & Kwon, 2023). However, results from nine studies presented null or contradictive evidence in their analysis of predictions following the SLT on the cycle of IPV perpetration (see. Craft & Serovich, 2005; Eriksson & Mazerolle, 2015; Forke et al., 2018; Franklin et al., 2012; Fritz et al., 2012; Haselschwerdt et al., 2021; Menard et al., 2014; Nikulina et al., 2021; Wareham et al., 2009). Craft and Serovich (2005) on the other hand found differences in the transmission pathways based on the type of violence behavior witnessed and the cumulative experiences. Compared to exposure to non-physical IPV alone, children who were abused in conjunction with witnessing parental violence (ibid) had higher odds of perpetrating IPV in their adult relationships. These findings were consistent with other studies in Canada and the United States (Eriksson & Mazerolle, 2015; Forke et al., 2018; Shields et al., 2020) which found a positive relationship between the cumulative effects of experiencing child abuse and exposure to parental violence in predicting future IPV perpetration outcomes.

Typically noted alongside SLT, the intergenerational transmission of violence (IGTV) hypothesis suggests that patterns of abusive behaviors and victimization are learned and perpetuated across generations. Fritz et al. (2012) found a strong association between exposure to parental violence and IPV perpetration, with coefficients suggesting that exposure to parental violence could increase risk of IPV perpetration by 39% to 40%. Thus, providing support for the intergenerational transmission hypothesis. Support for this theory-driven hypothesis was also found in twelve studies from seven countries (U.S., Canada, Peru, Thailand, Africa, Asia, and South Korea) (*cf.* Franklin et al., 2012; Gover et al., 2008; Haselschwerdt et al., 2021; Hernández & Durán, 2021; Heyman & Smith Slep, 2002; Jung et al., 2019; Kerley et al., 2010; Kernsmith, 2006; Kwon & You, 2023; Kwong et al., 2003; Mehfooz et al., 2023; Ruel et al., 2020; You & Kwon, 2023) with relatively consistent evidence. Specifically, a positive relationship between childhood exposure to IPV and IPV perpetration as an adult was found in a study conducted by Kerley et al. (2010) in Thailand. However, gender differences revealed nuances that highlight the complexities of the intergenerational transmission of IPV (*cf*. Fritz et al., 2012; Kerley et al., 2010). Wareham et al. (2009) noted the validity of the intergenerational theory, however, they argued that without the inclusion of other theoretical frameworks (e.g., SLT) it does not adequately explain the transmission of IPV.

Guided by psychosocial theory, Fergusson et al. (2006) found no significant evidence of the association between experiencing adversities, such as family dysfunction (witnessing violence) and greater socioeconomic disadvantage in childhood, and the likelihood of IPV perpetration in adulthood. However, two studies conducted in Canada, and the United States reported consistent findings on the transmission of IPV following prepositions from the ecological theory (Craft & Serovich, 2005; Shields et al., 2020). Men who experienced childhood physical abuse were more likely to express physically abusive violent behaviors in their intimate relationships (Craft & Serovich, 2005), and the relationship between childhood abuse history and IPV perpetration was found to be stronger due to victims’ employment status, and lack of access to affordable accommodation (Shields et al., 2020). In a similar vein, Knights’ et al. (2016) study following the interactional theory found statistically significant results on the association between exposure to IPV and IPV perpetration in the subsequent generation. However, the intergenerational perpetration of IPV was much stronger among female adult children compared to males (ibid).

#### Psychological Theories

Psychological theories such as attachment, general strain theory, and trauma theory were used as theoretical frameworks within six (20%) of the 30 included studies to explain the cycle of violence. Four studies were informed by attachment theory suggesting that a lack of parental attachment or decreased support during adolescence may increase the risk for combined victimization or perpetration for children who witnessed violence in the family-of-origin (Forke et al., 2018). Insecure attachment to parental figures that expressed rejection, a lack of care, and shaming by parents were positive predictors of future IPV perpetration and higher levels of attachment insecurity over the life course (*cf*. Antle et al., 2020, Sokar et al., 2023). A comparative study by Forke et al. (2021) in the United States found that children who witness IPV were more likely to experience victimization and perpetration in adolescence compared to their non-witness counterparts.

Menard et al.s’ (2014) study followed predictions from the general strain theory, whereas the trauma theory underpinned the study by Cheung and Huang (2023) in the United States. General strain theory (Agnew & White, 1992) posits that exposure to specific sources of stressors (e.g., witnessing parental violence) provokes a negative emotional response (e.g., anger, frustration, or disappointment) leading to coping through violent or criminal behaviors. Such mechanisms influence the use of violence in future relationships. Menard et al. (2014) hypothesized that witnessing parental violence is associated with adult IPV perpetration, however, this hypothesis was not supported and no significant relationships between the variables were found. Guided by the trauma theory, Cheung and Huang (2023) hypothesized that childhood exposure to IPV would increase adolescent involvement in TDV. Trauma theory postulates that unstable environments disrupt the sense of safety, intimacy, and trust, resulting in a disconnection from others and psychological vulnerabilities (Herman, 1992). Such vulnerabilities create enabling environments for IPV perpetration. Cheung and Huang’s (2023) study using a multivariate regression analysis strategy found a positive relationship between involvement in TDV and the cumulative effects of early exposure to IPV.

#### Cultural Factor

Four (13%) of the included studies examined cultural differences in exposure to IPV and subsequent victimization and/or perpetration. Kerley et al. (2010) examined the relationship between exposure to family violence and IPV in adulthood in Urban Thailand. Family structures within Thai culture have historically conformed to patriarchal influence, finding that Thai women exposed to IPV in childhood were more likely to be physically abused in adulthood. Similarly, Mehfooz et al. (2023) in their cross-continental cohort study found that Asian women were more likely to accept or justify partner violence, whereas African women were more likely to justify IPV for refusing sex, neglecting children, going out without telling their spouse, or arguing. Research conducted by Sokar et al. (2023) concluded that endorsement of patriarchal ideology contributed to the justification of wife beating in their Israeli sample and found that higher rates of witnessing IPV significantly correlated with IPV perpetration. Nam and Maxwell (2021) corroborated this evidence in their South Korean sample finding that witnessing parental conflict was linked to experiencing dating violence.

#### Gender and Role-Specific Modeling

A common theme noted among studies was the consideration of gender and role-specific modeling in the intergenerational transmission of IPV. Often applied alongside SLT, gender, and role-specific modeling suggests that certain characteristics, behaviors, and roles individuals take on are influenced by gender. While SLT postulates that violence is a learned behavior (Bandura, 1977), analysis of the influencing factor of gender assumes that behavior of the same-sex parent is imitated (Eriksson & Mazerolle, 2015). Thus, for example, male children who witness father-to-mother violence go on to perpetrate violence in their own intimate relationships.

Sokar et al. (2023) found a significant positive relationship between men who witnessed interparental IPV during childhood and IPV perpetration in adulthood, compared to women. Three studies from the United States (Forke et al., 2018, 2021; Heyman & Smith Slep, 2002) found similar results supporting IPV exposure increasing the likelihood of IPV perpetration for men, and higher victimization rates for exposed women. Yet, through structural equation modeling (SEM), Kwon and You (2023) found that men who witnessed and experienced maternal abuse were more likely to experience victimization in intimate relationships.

Contradictive to the aforementioned studies, only partial support was found for gender and role-specific modeling by Eriksson and Mazerolle (2015) who found that only witnessing bidirectional violence was predictive of IPV perpetration in adulthood. Gover et al.’s (2008) study concluded that witnessing abuse perpetrated by fathers significantly impacts females’ rates of victimization. Three studies, however, did not find significant statistical evidence to support the gender-specific hypothesis (Jung et al., 2019; Kwong et al., 2003; Menard et al., 2014).

## Discussion

The cybernetic relationship between theory, empirical research, and practice, is acknowledged within the research literature. This interconnected relationship highlights the relevance of theory in empirical research, such as research that offers insight into the pathways of IPV transmission from G1 to G2. This systematic review synthesized existing research on IPV transmission in the context of the theories tested in the empirical research. It further analyzed the nuanced cultural factors that influence the intergenerational transmission of IPV. The results, which have been presented under broad general theories, have been discussed in the context of theory-driven empirical research, and the shortfalls of theory application.

### Theory-Driven Research: Theoretical Postulations and How They Informed IPV Research

This review shows that the majority of research on the cycle of IPV transmission, from G1 to G2, is informed by predictions from SLT (Bandura, 1977). Specifically, following the SLT theory’s postulations on behavioral modeling, authors in these studies examined the propensity of IPV perpetration among young people who experienced/witnessed IPV in their childhood. Although almost half of the studies found significant statistical evidence to support their hypothesis (*cf.* Cheung & Huang, 2023; Forke et al., 2021; Karlsson et al., 2016; Kerley et al., 2010; Kernsmith, 2006; Knight et al., 2016; Kwon & You, 2023; Kwong et al., 2003; Mehfooz et al., 2023; Morris et al., 2015; Nam & Maxwell, 2021; Ruel et al., 2020; Sokar et al., 2023; You & Kwon, 2023), others reported null findings or contradictory evidence (*cf.* Craft & Serovich, 2005; Eriksson & Mazerolle, 2015; Forke et al., 2018; Franklin et al., 2012; Fritz et al., 2012; Haselschwerdt et al., 2021; Menard et al., 2014; Nikulina et al., 2021; Wareham et al., 2009). On the surface, the utilization of SLT theory in IPV research corroborates claims that the theory is useful for learning about behavioral modeling and transmission of observed behaviors (Copp et al., 2019; Forke et al., 2018; Valgardson & Schwartz, 2019). Much like studies that were informed by the SLT theory, studies that followed the IGTV theory’s postulations on violence transmission also reported similar patterns of results regarding the consistency and inconsistency of empirical evidence (*cf*. Franklin et al., 2012; Gover et al., 2008; Haselschwerdt et al., 2021; Hernández & Durán, 2021; Heyman & Smith Slep, 2002; Jung et al., 2019; Kerley et al., 2010; Kernsmith, 2006; Kwon & You, 2023; Kwong et al., 2003; Mehfooz et al., 2023; Ruel et al., 2020; You & Kwon, 2023).

Analytically, the mixed findings reported in these studies raise concerns about how the theories are applied and the usefulness of general theories against middle-range or micro-theoretical frameworks (Crothers, 2004; Merton, 1949). While Bandura’s (1977) SLT fits into the category of general theories (Merton, 1973), Akers (1977) version of the theory provides discrete variables, such as differential association, differential reinforcement, imitation, and modeling, which may fit into the category of a middle-range theory. Hence, to fully apply the SLT theory to examine IPV transmission intergenerationally, it is expected that the effects of the key constructs from the theory (imitation of observed behavior, behavioral modeling, and reinforcement) would be examined empirically to demonstrate their effects on the transmission. Otherwise, it begs the question to believe that a child who witnessed IPV during their childhood perpetrated IPV in his/her adulthood because he/she mimicked or imitated the behavior observed in their childhood. It may well be the case that the violence was reproduced in the child’s adult relationship due to the insecure attachment relationship he/she experienced, as per attachment theory, (*cf*. Antle et al., 2020, Sokar et al., 2023), or due to the child’s inability to cope with the stressful IPV experience, as per the general strain theory (Agnew & White, 1992). The true transmission pathway can be fully evaluated in studies that examine the key constructs from the theory and their impact on IPV transmission through a sophisticated statistical approach, such as mediation or moderation analysis, instead of a simple direct association.

Evidence from some of the included studies justifies the call for the examination of theory-driven variables in analyzing the transmission of IPV. Guided by attachment theory, the research of Forke et al. (2018) revealed the impact of insecure attachment, lack of care, and shaming by parents as among the key variables that impact IPV perpetration in adulthood. Such evidence provides concrete insight into how IPV transmits intergenerationally and offers areas/targets to implement interventions that address IPV transmission. For example, an intervention could be developed to provide adequate care and secure attachment to children who are exposed to IPV as part of the measures to prevent the transmission of IPV.

The lack of examination of the theory-driven variables, as evident in many of the reviewed studies, renders the theory application reductionist. Instead, what is evident across the included studies is what researchers describe as (a) secondary analysis, and (b) post factum (Boudon, 1969; Portes, 1971). In secondary analysis and post factum, researchers generate interpretations of null findings or counter-predictive findings within the same theory that informed the study (Merton, 1973). It is therefore not surprising that in the majority of the studies that generated counter-predictive findings, the authors justified the findings within the same theoretical framework, and supported such justifications with methodological and contextual limitations (Craft & Serovich, 2005; Eriksson & Mazerolle, 2015; Forke et al., 2018; Gover et al., 2008; Shields et al., 2020). For example, Craft and Serovich (2005) justified their null findings within the same theoretical framework by arguing that IPV transmission may well occur in specific types of violent behaviors, such as sexual coercion. Such scenarios may limit the advancement of science and theory development in relation to IPV transmission (Merton, 1973). Instead, it is recommended that the generation of counter-predictive evidence in empirical research offers an opportunity to rethink and reformulate theories, and in some cases formulate new theories that advance knowledge, since empirical research forms a core base for formulating theories (Hedström & Swedberg, 1996; Merton, 1948). For example, the inconsistent findings from studies across different countries on the SLT theory in the context of IPV (Craft & Serovich, 2005; Eriksson & Mazerolle, 2015; Forke et al., 2018; Franklin et al., 2012; Fritz et al., 2012; Haselschwerdt et al., 2021; Menard et al., 2014; Nikulina et al., 2021; Wareham et al., 2009) could have offered an opportunity for theoretical analysis and theory formulation, instead of secondary analysis.

Also evident from the included studies is the simplistic application of the theories. Hypotheses in all the included studies were examined following postulations from single psychological, or social theories. Yet, a plethora of research (Muniz & Zavala, 2023; Smith-Marek et al., 2015; Weir et al., 2021), including a systematic review (Kimber et al., 2018) have underscored the complex processes involved in the transmission of IPV. Such complexity is yet to be acknowledged theoretically as the included studies are limited by the fact that they resort to postulations from a single-arm of theory, instead of a multi-theory or complex theory-testing. For instance, it may well be the case that a child who is exposed to IPV may model IPV as acceptable behavior (SLT), however, if the same child received a secure attachment from the perpetrator and/or the non-offending parent (attachment theory) that may attenuate the likelihood of the child going on to perpetrate violence in their adult relationship. Similarly, a child may fail to cope with the stress and frustrations (general strain theory) and experience emotional difficulties (trauma theory) resulting from witnessing IPV, but the likelihood of such frustrations and emotional difficulties leading to IPV perpetration or victimization could be mitigated if the child develops in a supportive environment (ecological theory). Evidence from some of the included studies on the effects of child maltreatment and the link between exposure to IPV and later IPV perpetration highlight a potential case for multi-theory examination (Eriksson & Mazerolle, 2015; Forke et al., 2018; Shields et al., 2020), as it shows the impact of poly-victimization experiences on IPV transmission (Emery et al., 2022; Finkelhor et al., 2007). Studies that adopt a multi-theory or comprehensive approach to examining IPV transmission are likely to yield greater insights required to inform IPV research and intervention.

Further, findings on the impacts of cultural norms on IPV transmission, including patriarchal beliefs, across studies in Thailand, South Korea and other Asian countries (Kerley et al., 2010; Mehfooz et al., 2023; Nam & Maxwell, 2021; Sokar et al., 2023) exemplify the complexity of the processes involved in the transmission of IPV. Sokar et al. (2023) findings that patriarchal ideology enforced norms of wife beating and impacted the intergenerational transmission of IPV highlights the importance of social norms and social processes in enforcing IPV (*cf*. Capaldi et al., 2012; Yakubovich et al., 2018). Similarly, the findings suggest a nuanced relationship between gender, role-specific modeling, and IPV perpetration. Some of the included studies provided evidence supporting the notion of witnessing father-to-mother violence increasing the likelihood of male children becoming perpetrators of IPV (Forke et al., 2018, 2021; Heyman & Smith Slep, 2002). Others provided evidence suggesting this could happen through witnessing bidirectional IPV (Eriksson & Mazerolle, 2015). In addition to corroborating existing evidence (Hou et al., 2016) these findings suggest the need to adopt a comprehensive approach to study the transmission of IPV intergenerationally and the impact on victims.

### Implications for Research and Theory Development on IPV Transmission

Evidence from the included studies suggests that research on the intergenerational transmission of IPV may suffer from a reductionist application of theories. This limitation is present through the superficial application of theoretical models such as SLT and general strain theory, where researchers have consistently overlooked the key theory-driven constructs that could effectively explain IPV transmission pathways in their studies. Thus, the application of these theories is insufficient, limiting the ability to fully evaluate the mechanisms that undergird the transmission of IPV.

The lack of empirical examination of theory-drive variables may motivate researchers to “cherry-pick” theories or be involved in secondary analysis by seeking to provide logical interpretations to support counter-predictive evidence. Even for studies that find evidence to support their hypothesis, it becomes impossible to evaluate the variables or processes that underpinned the transmission. For example, a positive relationship between witnessing IPV as a child and perpetrating IPV as an adult could be explained within the frameworks of either SLT, trauma, IGTV, or general strain, if no theory-driven variables are tested. However, studies that examine the mediating effects of emotional responses to childhood stress, anger, and frustration from witnessing IPV, leading to IPV transmission may provide a concrete evaluation of the general strain theory in the context of IPV transmission. As a result, we recommend that further research be conducted specifically focusing on the theory-driven variables that are predicted to carry the transmission effects of IPV from childhood to adulthood.

**Figure 2.**
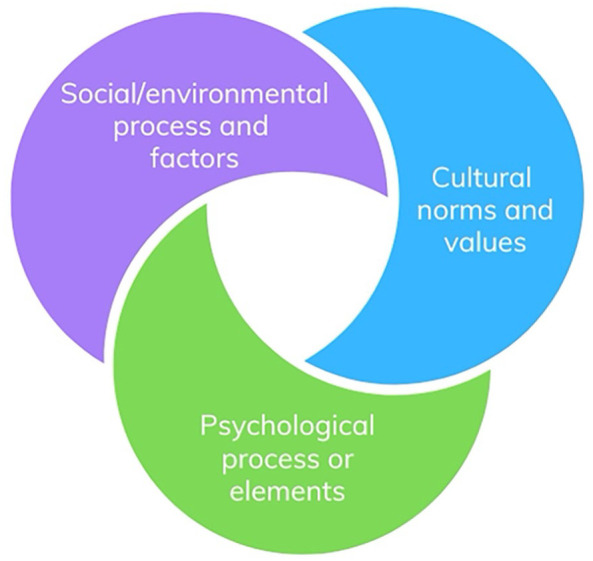
Integrated theoretical model.

Additionally, the study findings have evidenced the complex processes and factors that could shape the transmission of IPV. Specifically, it highlighted the social, psychological, behavioral, cultural, and identity factors that may interplay to motivate or deter IPV perpetration. A simple claim about IPV transmission via behavioral imitation may indeed be considered a façade and reductionist and risks overlooking the complexities of transmission. A reductive approach to IPV research may further support the misconceptions around IPV dynamics. Hence, we call for the development of a comprehensive theoretical framework that considers the nuances of social, cultural, and psychological processes and factors that regulate the transmission of IPV intergenerationally. A more holistic framework would ideally highlight the cybernetic and interactional relationship between these core areas (social, psychological, and cultural) in influencing IPV transmission. Such an approach would acknowledge the interconnected factors that influence the cyclic nature of IPV transmission across generations and operate from a more comprehensive framework than a single-arm theory can adequately capture. Figure 2 below represents the conceptual aspects of this potential framework.

### Limitations

Although the current study provides a synthesis of studies that examined the transmission of IPV intergenerationally, some limitations may apply to the study. First, the study is limited to articles retrieved from the databases included. It may be possible to have missed out on some studies that are not indexed in the targeted databases. Second, the study findings are limited to articles published in English after 1990. Studies published before 1990 and those published in other languages may include evidence that could have enriched the current findings. Third, although we analyzed the included studies based on the theories that informed the study hypothesis, we acknowledged that the consistency and inconsistency of quantitative evidence are also impacted by the study methodology. Hence, a methodological analysis of the included studies could complement the study findings. Fourth, our discussion of the consistency and inconsistency of evidence from the included studies is based on a narrative discussion of the quantitative evidence, in terms of the direction of the relationships and coefficient reported. A meta-analysis of the included studies using the same theoretical lens could provide additional evidence to complement the current findings.

### Conclusion

The processes involved in the transmission of IPV are undoubtedly complex. A significant amount of existing empirical research guided by theoretical postulations on IPV transmission pathways has found null or counter-predictive findings. This review analyzed evidence from the extant literature on the cycle of IPV transmission by focusing on the theories that underpinned the studies. The review revealed the need to adopt a comprehensive theoretical framework that better attempts to understand the multifaceted nature of IPV and considers the various interactional factors contributing to motivating or deterring IPV transmission. Future research focusing on theory-driven variables may provide a better insight into the transmission of IPV and the theoretical mechanisms that underpin the transmission process.
